# Draft Genome Sequence of *Staphylococcus epidermidis* (Winslow and Winslow) Evans (ATCC 14990)

**DOI:** 10.1128/genomeA.00619-17

**Published:** 2017-07-06

**Authors:** Catherine Putonti, Laurynas Kalesinskas, Evan Cudone, Kathleen C. Engelbrecht, David W. Koenig, Alan J. Wolfe

**Affiliations:** a Bioinformatics Program, Loyola University Chicago, Chicago, Illinois, USA; b Department of Biology, Loyola University Chicago, Chicago, Illinois, USA; c Department of Computer Science, Loyola University Chicago, Chicago, Illinois, USA; d Department of Microbiology and Immunology, Stritch School of Medicine, Health Sciences Division, Loyola University Chicago, Maywood, Illinois, USA; e Corporate Research and Engineering, Kimberly-Clark Corporation, Neenah, Wisconsin, USA

## Abstract

Here, we report the draft genome sequence for the type strain *Staphylococcus epidermidis* (Winslow and Winslow) Evans (ATCC 14990). The assembly consisted of 2,457,519 bp with an observed G+C content of 32.04%. Thirty-seven contigs were produced, including two putative plasmids, with a 296.8× coverage and an *N*_50_ of 180,848 bp.

## GENOME ANNOUNCEMENT

As part of an attempt to generate complete genomes for a subset of type strains in the ATCC collection, we report here the genome sequence and annotation of *Staphylococcus epidermidis* (Winslow and Winslow) Evans (ATCC 14990) isolated from the nose. 

The purchased culture isolate was grown on 5% sheep blood agar (BD BBL prepared plated media) under 5% CO_2_ at 35°C for 48 h. To extract genomic DNA, cells were resuspended in 0.5 mL DNA extraction buffer (20 mM Tris-Cl, 2 mM EDTA, 1.2% Triton X-100, pH 8), followed by the addition of 50 µL of lysozyme (20 mg/mL), 30 µL of mutanolysin, and 5 µL of RNase (10 mg/mL). After incubation at 37°C for 1 h, 80 μL of 10% SDS and 20 µL of proteinase K were added and incubated at 55°C for 2 h. Then, 210 μL of 6M NaCl and 700 μL of phenol-chloroform were added and incubated with rotation for 30 min, followed by a 10-min centrifugation at 13,500 rpm. The aqueous phase was extracted, and an equivalent volume of isopropanol was added. The solution was centrifuged at 13,500 rpm for 10 min after a 10-min incubation. The supernatant was decanted, and the DNA pellet was precipitated using 600 μL of 70% ethanol. Following ethanol evaporation, the DNA pellet was resuspended in Tris-EDTA and stored at −20°C.

The extracted genomic DNA was diluted in water to a concentration of 0.2 ng/µL, as measured by a fluorometric-based method (Life Technologies, Inc.). Library preparation of 1 ng (5 μL) of input DNA was performed using the Nextera XT DNA library preparation kit. The library was sequenced on the MiSeq sequencer (Illumina) using the MiSeq version 2 reagent kit (500 cycles) producing 1,689,436 paired-end reads in total. Reads were first processed, removing adapter sequences and phiX contaminants, using BBDuk from the BBMap package (http://sourceforge.net/projects/bbmap). The resulting trimmed reads were assembled using SPAdes version 3.5 (1), followed by scaffolding with SSPACE (2). In total, 37 contigs, varying in size from 504 bp to 749,904 bp (*N*_50_ = 180,848 bp), were produced with an average coverage of 296.8×. Two of these contigs (13,346 bp and 4,566 bp in length) correspond to individual plasmids within the strain. Confirmed via BLAST (BLASTn) to the GenBank NR/NT nucleotide database, these two plasmid sequences exhibit homology to *S. epidermidis* ATCC 12228 plasmid pSE-12228-04 (GenBank no. AE015933) and *S. aureus* plasmid SAP093A (GenBank no. GQ900441), respectively. Annotations were generated using the software tool Peasant (3). Nine rRNAs, 59 tRNAs, and 2,274 protein-coding sequences were identified. Furthermore, one possible clustered regularly interspaced short palindromic repeat (CRISPR) array was found (4). The final genome size for the *S. epidermidis* strain Evans (ATCC 14990) was 2,457,519 bp with an observed G+C content of 32.04%.

### Accession number(s).

The draft whole-genome project for *S. epidermidis* strain Evans (ATCC 14990) has been deposited at DDBJ/EMBL/GenBank under accession number NARC00000000. Raw sequence reads are deposited at DDBJ/EMBL/GenBank under accession number SRR5364302.
